# MICHELINdb: a web-based tool for mining of helminth-microbiota interaction datasets, and a meta-analysis of current research

**DOI:** 10.1186/s40168-019-0782-7

**Published:** 2020-02-03

**Authors:** Riccardo Scotti, Stuart Southern, Christine Boinett, Timothy P. Jenkins, Alba Cortés, Cinzia Cantacessi

**Affiliations:** 1 Department of Veterinary Medicine, University of Cambridge, Madingley Road, Cambridge, CB3 0ES UK; 2 Present address: Quadram Institute Bioscience, Norwich Research Park, Norwich, UK

## Abstract

**Background:**

The complex network of interactions occurring between gastrointestinal (GI) and extra-intestinal (EI) parasitic helminths of humans and animals and the resident gut microbial flora is attracting increasing attention from biomedical researchers, because of the likely implications for the pathophysiology of helminth infection and disease. Nevertheless, the vast heterogeneity of study designs and microbial community profiling strategies, and of bioinformatic and biostatistical approaches for analyses of metagenomic sequence datasets hinder the identification of bacterial targets for follow-up experimental investigations of helminth-microbiota cross-talk. Furthermore, comparative analyses of published datasets are made difficult by the unavailability of a unique repository for metagenomic sequence data and associated metadata linked to studies aimed to explore potential changes in the composition of the vertebrate gut microbiota in response to GI and/or EI helminth infections.

**Results:**

Here, we undertake a meta-analysis of available metagenomic sequence data linked to published studies on helminth-microbiota cross-talk in humans and veterinary species using a single bioinformatic pipeline, and introduce the 'MICrobiome HELminth INteractions database' (MICHELINdb), an online resource for mining of published sequence datasets, and corresponding metadata, generated in these investigations.

**Conclusions:**

By increasing data accessibility, we aim to provide the scientific community with a platform to identify gut microbial populations with potential roles in the pathophysiology of helminth disease and parasite-mediated suppression of host inflammatory responses, and facilitate the design of experiments aimed to disentangle the cause(s) and effect(s) of helminth-microbiota relationships.

Video abstract.

## Background

In the world, > 1.5 billion people are infected with parasitic helminths (worms), including the gastrointestinal (GI) roundworms *Trichuris trichiura*, *Ascaris lumbricoides*, *Necator americanus* and *Ancylostoma duodenale* and the extra-intestinal (EI) blood flukes *Schistosoma mansoni*, *S*. *japonicum* and *S*. *haematobium* [1–3]. In endemic areas of Africa, South America and South-East Asia characterised by poor sanitation and sub-optimal hygiene standards, these parasites are responsible for considerable morbidity and mortality, particularly in vulnerable groups such as children and pregnant women [4, 5]. Furthermore, GI and EI parasites of livestock are responsible for substantial economic losses worldwide, due to impaired production, growth retardation, treatment costs and/or stock replacement [6]. Currently, control of parasitic helminths relies heavily on the administration of anthelmintics *via* mass drug administration and targeted strategic treatment programmes in humans [7, 8] and veterinary species [9, 10], respectively. However, drug resistance to all available classes of anthelmintics is widespread in helminths of livestock [9, 10] and the threat of emerging anthelmintic resistance in human parasites is concrete [11]. In addition, even after successful elimination of worms, both humans and animals remain at-risk of re-infections, which often occur rapidly in endemic areas [12, 13]. Thus, the discovery and development of alternative strategies of helminth control has long been a major focus of the global ‘One Health’ agenda [14–16]. A thorough understanding of the fundamental biology of helminth parasites and of host-pathogen interactions may assist the search for new drug targets.

Parasitic helminths are long-lived in the vertebrate hosts and often establish infections without evoking symptomatic inflammatory reactions [17, 18]. The ability of parasites to manipulate the host immune system to their advantage is a key area of research in host-parasite relationships which, over the years, has led to the identification of a number of helminth excreted-secreted molecules with immune-modulatory properties [18, 19]. However, recently, evidence has started to emerge of another likely player in this paradigm, i.e. the host gut microbiota [20–22]. In particular, several studies have documented the impact that infections by GI and EI parasitic helminths exert on bacterial populations inhabiting the vertebrate gut, with likely downstream effects on digestion and nutrient absorption, immune and metabolic homeostasis and infection pathophysiology [23–28]. Nevertheless, whilst a small number of qualitative and/or quantitative changes in the composition of the host gut microbiota have been repeatedly observed in helminth-infected humans or animals and irrespective of infecting helminth species, the vast majority of published investigations are characterised by inconsistent and seldom contradictory findings [20, 29]. These apparent discrepancies may be linked to the fundamentally diverse biology of the parasites under investigation, that might result in microbiota alterations that are specific to the colonising helminth species. However, one possible explanation is technical and linked to the vast heterogeneity of experimental designs and metagenomic sequence analysis techniques that characterise currently available studies and that may have contributed to the observed differences between published datasets [29].

Similarly, the availability of a range of bioinformatic pipelines and reference databases for metagenomic data analysis and annotation, each with its *pros* and *cons*, may substantially impact the final outputs, thus making findings from studies utilising different workflows not directly comparable. Whilst a complete standardisation of study designs and experimental protocols is difficult to achieve, bioinformatic (re)analyses of sequence data using a single workflow and up-to-date databases for sequence annotation may assist the detection of common sets of findings across studies [29]. Such knowledge is indeed crucial to evaluate the real impact (if any) of helminth infections on the composition and the metabolic functions of the host gut microbiota, and thus to develop strategies to minimise such effects.

Thus far, the vast majority of published studies on helminth-microbiota interactions have relied on high-throughput sequencing of the bacterial 16S rRNA gene fragment for determination of host gut microbial profiles prior to and following helminth infections [20]; these studies will represent the focus of the present article. Amongst the bioinformatic tools for sequence analysis and annotation, the Quantitative Insights Into Microbial Ecology (QIIME) [30] and Mothur [31] pipelines have been the most widely used platforms for taxonomic analyses of metagenomic sequence data generated in these experiments; nevertheless, recent benchmarking tests have shown that other open source software, such as Multiplexed Analysis of Projections by Sequencing (MAPseq) [32] and QIIME 2 (an updated version of QIIME) [33] provide a faster and more accurate read classification, better sensitivity and specificity at different taxonomic levels and miscall rates of < 2% [34].

Furthermore, the accurate annotation of bacterial 16S rRNA sequence data relies on the availability of continuously updated and curated reference databases; thus far, the Greengenes database (http://greengenes.secondgenome.com/) has been widely used in bioinformatic analyses of sequence data generated in studies of helminth-microbiota interactions [28, 35, 36]. However, the last update of this database dates back to 2013, and therefore it is likely that sequence information available from this repository are incomplete and/or outdated. Conversely, the SILVA database (https://www.arb-silva.de/) [37] is frequently updated (latest release: December 2017) and comprehensive, as it also includes annotated datasets of aligned rRNA sequences for Bacteria, Archaea and Eukarya [37, 38].

In this study, we undertake a meta-analysis of high-throughput amplicon 16S rRNA sequence datasets linked to studies of helminth-microbiota interactions in humans and veterinary species, using consistent data reprocessing; in addition, in order to facilitate the retrieval of information generated in published investigations conducted in different host-parasite pairs, under varying conditions of helminth infection (i.e. natural *vs*. experimental and acute *vs*. chronic), and analysing different biological specimens (i.e. faeces *vs*. host GI tissue samples), we created the 'MICrobiome HELminth INteractions database' (MICHELINdb; www.michelindb.com). In this database, information generated from individual studies of helminth-microbiota interactions are stored together with associated metadata on host and parasite species, infection site, sample type and metagenomic sequencing strategy (amongst others), and links to raw sequence data and published papers. By increasing the accessibility of these datasets and by presenting the results of a meta-analysis of available data using a single bioinformatic pipeline, we aim to provide the scientific community with a platform to identify gut microbial signatures that occur across several host-helminth systems, and facilitate the design of experiments aimed to disentangle the cause(s) and effect(s) of helminth-microbiota relationships.

## Materials and methods

### Study selection and data acquisition

Published studies on the composition of the gut microbiota of vertebrate hosts acutely and/or chronically infected by parasitic helminths (nematodes, cestodes and trematodes) were retrieved by mining search engines (i.e. NCBI PubMed, ISI Web of Knowledge, Google Scholar and ScienceDirect) using the terms ‘gut’ AND/OR ‘microbiome’ AND/OR ‘microbiota’ AND/OR ‘parasite’ AND/OR ‘helminth’ and by following references in published reviews and related studies. All sequence datasets retrieved using this method, as well as associated metadata, were included in MICHELINdb, together with study findings (see below), whilst only studies that satisfied the following criteria were included in the meta-analysis:

- Available paired-end high-throughput bacterial 16S rRNA sequence data in .fastq format deposited in publicly accessible databases (e.g. the NCBI Sequence Read Archive [SRA] or EBI European Nucleotide Archive [ENA])
- Available metadata on matching helminth-infected and -uninfected biological specimens and specimen origin (e.g. small or large intestine, faeces, etc.)

**Fig. 1:**
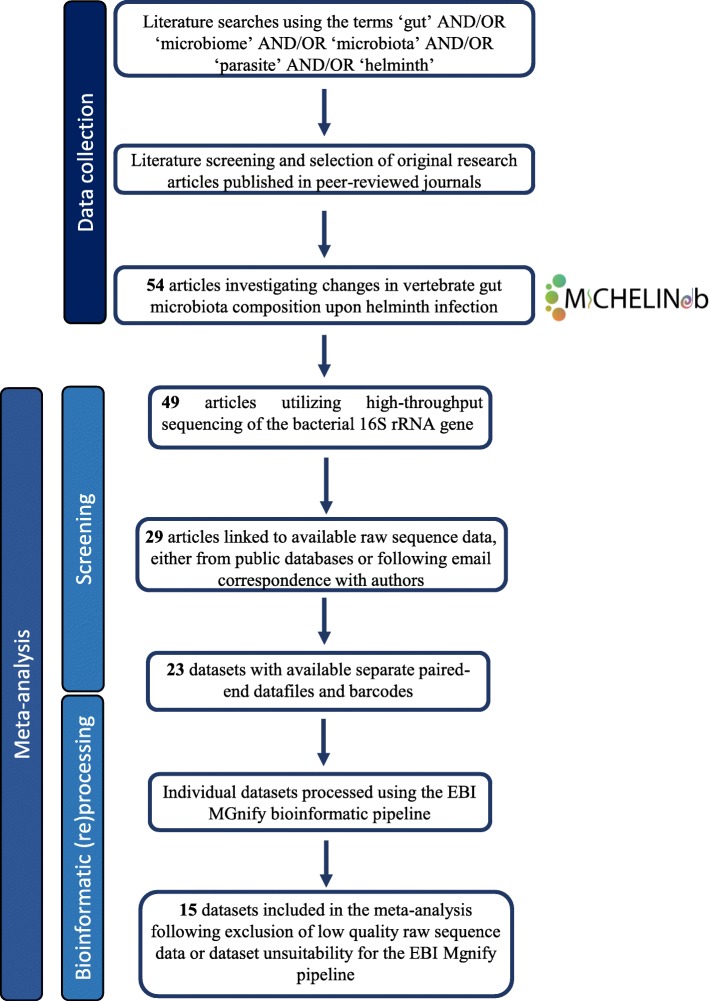
Flow diagram depicting the selection of studies for inclusion in the meta-analysis

Sequence datasets linked to studies that required additional ethics committee approval or institutional authorization for data access were not included in the meta-analysis; however, these datasets and corresponding findings, as presented in the original publications, are available for mining in MICHELINdb. For studies investigating the impact of anthelmintic administration on host gut microbiota composition, only sequence data obtained from biological specimens collected from helminth-infected and -uninfected human or animal controls were included in the meta-analysis; nevertheless, any available information on observed differences in microbial composition between specimens collected prior to and following anthelmintic treatment (as per original publication) is included in MICHELINdb. Biological specimens collected from the ileum and duodenum of vertebrate hosts were classified as originating from the small intestine. A summary of the publication selection strategy adopted in our meta-analysis is available from Fig. 1.

### Data processing

Raw sequence data representing the hypervariable regions of the bacterial 16S rRNA gene and linked to the studies outlined above were obtained from public sequence data repositories using accession numbers provided in the corresponding publications or following direct email communication with the corresponding authors (Table 1). When needed, provided barcodes were matched to sequences for demultiplexing. Then, sequence data were (re)processed (individually for each study) using the European Bioinformatics Institute (EBI) MGnify service pipeline, that integrates MAPseq v 1.2 [32] for operational taxonomic unit (OTU) assignment and SILVA (https://www.arb-silva.de/) [37] as the reference database for taxonomic classification. For details on the full properties of the MGnify pipeline, its performance compared to other open source software for high-throughput 16S rRNA sequence data analysis, as well as on its validation for metagenomic studies, we refer to the original publication by Mitchell et al. [50]. For each study included in the meta-analysis, OTU assignments are available from EBI MGnify (https://www.ebi.ac.uk/metagenomics/), under the corresponding MGnify/ENA accession reported in Table 1. For downstream analyses, samples containing < 100 reads were removed and OTU and taxonomy tables from each sample were merged using the feature-table merge command in QIIME 2 [33]. OTUs represented by < 10 reads and/or making up < 2% of the total number of OTUs identified in a given sample, as well as OTUs assigned to chloroplast taxa, were removed prior to statistical analyses.

**Table 1:** Studies from which bacterial 16S rRNA sequence datasets included in our meta-analysis were sourced, in chronological order. For each study, bibliographical references (including PubMed ID; PMID), as well as helminth parasite(s) and vertebrate host species under investigation, sequence data repository and corresponding accession number, are provided. The numbers of samples from helminth-infected and -uninfected hosts from each study included in the meta-analysis, and of raw and high-quality reads subjected to (re)processing are also included. Results from dataset (re)processing using MGnify are available in ENA MGnify (https://www.ebi.ac.uk/metagenomics/) using the accession numbers provided

PMID	Ref.	Host	Parasite	Type of infection	Type/site of sampling	16S rRNA hypervariable region	Pipeline	Ref. DB	ENA Accession	MGnify accession	Sample size		Raw read count		High-quality read count	
											Uninfected	Infected	Uninfected	Infected	Uninfected	Infected
23925654	[39]	Hamster (*Mesocricetus auratus*)	*Opisthorchis viverrini*	Experimental	Colon	V7-V9	CloVR-16S	RDP	PRJNA188112	MGYS00003431	4	4	179,193	189,399	149,780	157,744
24795483	[40]	Human (*Homo sapiens*)	*Necator americanus*	Experimental	Faeces	V1-V3 V3-V5	QIIME	Greengenes	PRJNA244913	MGYS00003191	8	8	106,832	103,428	86,737	83,780
26377648	[25]	Mouse (*Mus musculus*)	*Nippostrongylus brasiliensis*	Experimental	Small intestine	V3-V4	QIIME	Greengenes	PRJNA255974	MGYS00003192	10	9	77,836	71,752	77,836	71,752
25942314	[41]	Mouse	*Trichuris muris*	Experimental	Caecum, faeces	V4	QIIME	Greengenes	PRJEB6560	MGYS00001732	80	60	3,253,762	2,438,665	3,034,081	2,306,376
26150661	[35]	Wild yellow-necked mouse (*Apodemus flavicollis*)	Multiparasites	Natural	Stomach, small intestine, caecum, colon	V1-V3	QIIME	Greengenes	PRJEB8312	MGYS00003181	38	93	1,790,824	4,990,294	1,723,425	4,865,341
25934152	[42]	Cat (*Felis catus*) Dog (*Canis lupus familiaris*)	Multiparasites	Natural	Faeces	V4	MR DNA	Greengenes	PRJNA276586	MGYS00003432	14	48	587,641	2,002,497	500,349	1,657,742
27912797	[43]	Cat	*Toxocara cati*	Natural	Faeces	V3-V4	QIIME	Greengenes	PRJNA349988	MGYS00003433	21	24	4,121,930	6,206,792	3,365,062	4,999,740
27827438	[44]	Human	*N. americanus*	Experimental	Small intestine	V3-V4	QIIME	Greengenes	PRJNA316208	MGYS00003180	12	12	7,855,514	6,597,567	7,025,186	5,918,376
26853110	[36]	Alpine goat (*Capra aegagrus hircus*)	*Haemonchus contortus*	Experimental	Abomasum	V3-V4	QIIME	Greengenes	PRJNA518079	MGYS00004556	5	12	1,213,261	3,302,574	1,196,878	3,263,552
28892494	[45]	Human	Multiparasites	Natural	Faeces	V3-V4	QIIME	Greengenes	PRJEB21999	MGYS00003147	22	11	8,388,179	4,934,705	7,777,849	4,506,626
30353019	[46]	Human	*Strongyloides stercoralis*	Natural	Faeces	V3-V4	QIIME2	Greengenes	PRJEB28700	MGYS00003148	11	20	1,946,889	2,578,359	1,873,123	2,487,145
30104612	[28]	Mouse	*Schistosoma mansoni*	Experimental	Small and large intestine	V4	QIIME	Greengenes	PRJEB29315	MGYS00003460	40	40	1,405,122	1,369,830	1,405,120	1,369,830
30149004	[47]	Mouse	*Metagonimus yokogawai*	Experimental	Caecum	V3-V4	Mothur	EzBioCloud	PRJNA481634	MGYS00003893	7	8	3,335,612	1,405,858	3,194,586	1,351,499
30323795	[48]	Human	*Clonorchis sinensis*	Natural	Faeces	V3-V4	QIIME	Greengenes	PRJNA486437	MGYS00003894	42	47	5,010,319	5,131,918	4,814,736	4,997,256
30643702	[49]	Human	Multiparasites	Natural	Faeces	V4	Mothur	RDP	PRJNA487588	MGYS00003908	6	16	88,768	338,701	86,195	328,849

### Statistical analyses

All statistical analyses were executed using both the Calypso software (cgenome.net/calypso/) [51] and a custom-made code in R statistical software version 3.5.2, packages ‘phyloseq’, ‘vegan’, ‘meta’, ‘metaphor’ and ‘ggplot2’.

For microbial diversity indices calculation, sequence data were rarefied to the highest sequencing depth required to retain all study samples. Then, differences in bacterial alpha diversity (Shannon index), and evenness between the microbiota sequence data generated from helminth-infected and corresponding uninfected specimens were calculated. To produce a robust and conservative estimation of alpha diversity indexes, whilst accounting for the heterogeneity of data across studies, a random-effects meta-analysis model with DerSimonian–Laird estimator, adjusted with the Hartung-Knapp-Sidik-Jonkman method, was used to pool effect sizes and corresponding standard deviations from all of the studies included in our meta-analysis.

Microbial profiles of individual specimens were clustered at OTU level using principal coordinates analysis (PCoA), based on Bray-Curtis dissimilarity matrices and with ‘host’ and ‘sampling site’ as explanatory variables. In order to assess associations between the microbial profiles of helminth-uninfected and -infected specimens and parasite infections, a supervised canonical correspondence analysis (CCA) with ‘infection status’ as explanatory variable, was applied. The average relative abundance of bacterial phyla and genera was calculated as mean across all ‘infected’ and ‘uninfected’ specimens.

The interpolated area under the receiver operating characteristic (ROC) curve (= AUC) was used to identify bacterial taxa associated with host infection status. Briefly, ‘infection status’ and ‘microbial relative abundance’ were used as explanatory variables and outcome, respectively, in a random forest (RF) statistical classifier. A predicted mean decreasing accuracy (MDA) score was then generated for each taxon, at phylum and genus level, using the estimated RF model; subsequently, taxa were classified using AUC > 0.5 as cut-off. In comparative analyses between gut microbial profiles of helminth-infected and -uninfected hosts, only phyla making up > 1% of the overall microbiota, and genera > 0.5% in at least one group were considered (Table 2).

**Table 2:** Bacterial taxa associated with host infection status, at phylum and genus level, identified using the interpolated area under the receiver operating characteristic (ROC) curve (= AUC) in a random forest (RF) classifier. The predicted mean decreasing accuracy (MDA) score generated for each taxon, using the estimated RF model, is also shown. AUCs > 0.5 are not shown. In comparative analyses between gut microbial profiles of helminth-infected and -uninfected hosts, only phyla and genera making up > 1% and > 0.5% of the overall microbiota, respectively, in at least one group are reported

		Relative abundance (%)			
Phylum	Genus	AUC	Fold change	Uninfected	Infected
*Non-rodents*					
Actinobacteria	*Collinsella*	0.56	− 0.64	2.37	3.69
Actinobacteria	*Bifidobacterium*	0.54	0.85	2.68	2.26
Bacteroidetes		0.58	1.18	18.83	16.01
	*Bacteroides*	0.60	0.55	6.97	3.83
	*Parabacteroides*	0.58	0.71	0.51	0.37
	*Alloprevotella*	0.51	0.69	1.05	0.72
Firmicutes		0.56	− 1.10	48.94	54.05
	*Streptococcus*	0.52	0.65	2.58	1.69
	*Catenibacterium*	0.53	− 0.41	0.33	0.80
	*Holdemanella*	0.57	− 0.51	0.76	1.48
	*Lachnoclostridium*	0.52	0.98	0.58	0.57
	*Subdoligranulum*	0.55	0.83	2.39	1.99
	*Veillonella*	0.54	− 0.46	0.29	0.62
	*Peptoclostridium*	0.55	− 0.48	0.84	1.76
	*Blautia*	0.54	− 0.81	1.77	2.19
	*Megamonas*	0.54	− 0.45	0.35	0.78
	*Romboutsia*	0.52	− 0.72	0.65	0.89
	*Faecalibacterium*	0.55	0.88	2.64	2.32
	*Dialister*	0.54	0.78	0.97	0.76
	*Turicibacter*	0.52	− 0.29	0.16	0.56
Fusobacteria		0.55	− 1.21	1.70	2.06
	*Fusobacterium*	0.57	− 0.91	1.60	1.77
*Rodents*					
Actinobacteria		0.54	1.33	1.27	0.95
	*Bifidobacterium*	0.62	1.66	0.63	0.38
Bacteroidetes		0.68	1.51	46.02	30.4
	*Alistipes*	0.57	− 1.08	1.66	1.79
	*Bacteroides*	0.61	− 1.06	1.06	1.13
	*Odoribacter*	0.57	− 1.5	0.53	0.8
Firmicutes		0.65	− 1.25	48.33	60.46
	*Turicibacter*	0.58	5.44	1.83	0.34
	*Lactobacillus*	0.69	− 2.2	15.37	33.82
	*Faecalibaculum*	0.62	1.98	3.01	1.52
	*Dubosiella*	0.61	2.97	1.24	0.42
	*Candidatus Arthromitus*	0.62	− 59.75	0.01	0.74
	*Roseburia*	0.59	1.05	0.63	0.6
Proteobacteria	*Desulfovibrio*	0.62	1.94	0.64	0.33
	*Helicobacter*	0.57	− 7.69	0.1	0.74
Verrucomicrobia		0.58	− 3.09	0.7	2.16
	*Akkermansia*	0.59	− 3.14	0.68	2.14

### Database design

MICHELINdb was constructed as a relational database application to facilitate mining of information linked to available bacterial 16S rRNA sequence datasets generated in published studies aimed to uncover associations between GI and EI helminth infections and host gut microbiota composition. MICHELINdb was implemented using the MSSQL Express software (https://www.microsoft.com/en-au/sql-server/sql-server-editions-express), accessed via an N-Tier architecture (written in C# and ASP.NET MVC) on which the database (http://www.michelindb.com) resides.

## Results

### Selected studies on helminth-microbiota interactions and corresponding bacterial 16S rRNA sequence datasets

Literature database searches using the keywords listed above yielded 54 unique studies, published between 2010 and 2019, on the interactions between GI and EI helminth parasites and the gut microbiota of their vertebrate hosts (Additional file 1: Table S1). Of these, 15 matched the criteria outlined above and were retained in our meta-analysis (Table 1). These studies spanned ten helminth species, including nematodes (e.g. *N*. *americanus* and *Haemonchus contortus*) and trematodes (e.g. *Opisthorchis viverrini* and *S*. *mansoni*), of both public health and veterinary significance, and seven vertebrate hosts (i.e. human, cat, dog, goat, hamster, mouse and wild-mouse); of 15 studies included in our meta-analysis, 14 targeted the V3-V4 hypervariable region of the bacterial 16S rRNA gene (Table 1). Sequence data from a total of 732 biological specimens (i.e. faecal or tissue samples) were included in the meta-analysis, of which 413 were annotated as originating from helminth-infected hosts, and 319 from matching uninfected controls (Table 1 and Additional file 2: Table S2). A total of 80,924,051 paired-end reads were collected and subjected to (re)processing, of which 41,562,339 and 39,361,712 represented sequences generated from helminth-infected and -uninfected biological specimens, respectively (Table 1 and Additional file 2: Table S2). Following trimming and quality filtering, a total of 74,676,551 high-quality reads (i.e. 38,365,608 and 36,310,943 generated from helminth-infected and -uninfected specimens, respectively) were retained for downstream analyses (Table 1 and Additional file 2: Table S2).

### Clustering of microbial communities

**Fig. 2:**
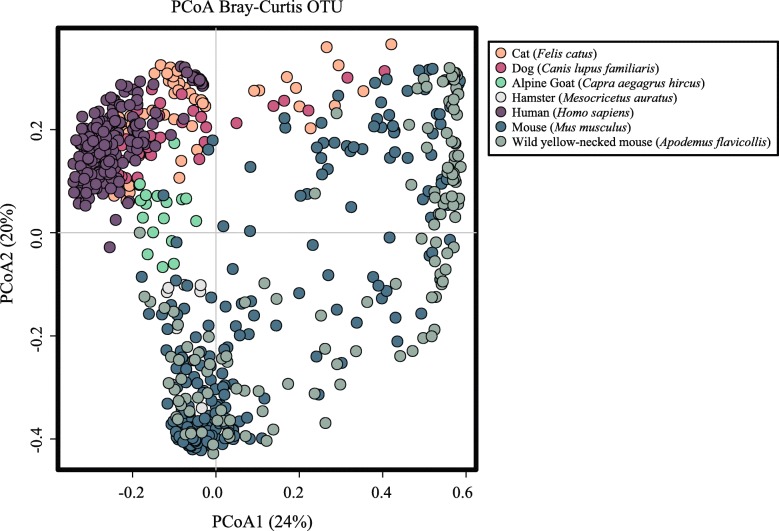
Gut microbial profiles of individual biological specimens (dots) from helminth-infected and -uninfected *Non-rodents* (i.e. human, cat, dog and goat) and *Rodents* (i.e. mouse, hamster and wild yellow-necked mouse) included in the meta-analysis, ordinated at operational taxonomic unit (OTU) level by principal coordinates analysis (PCoA), using ‘host’ as explanatory variable

**Fig. 3:**
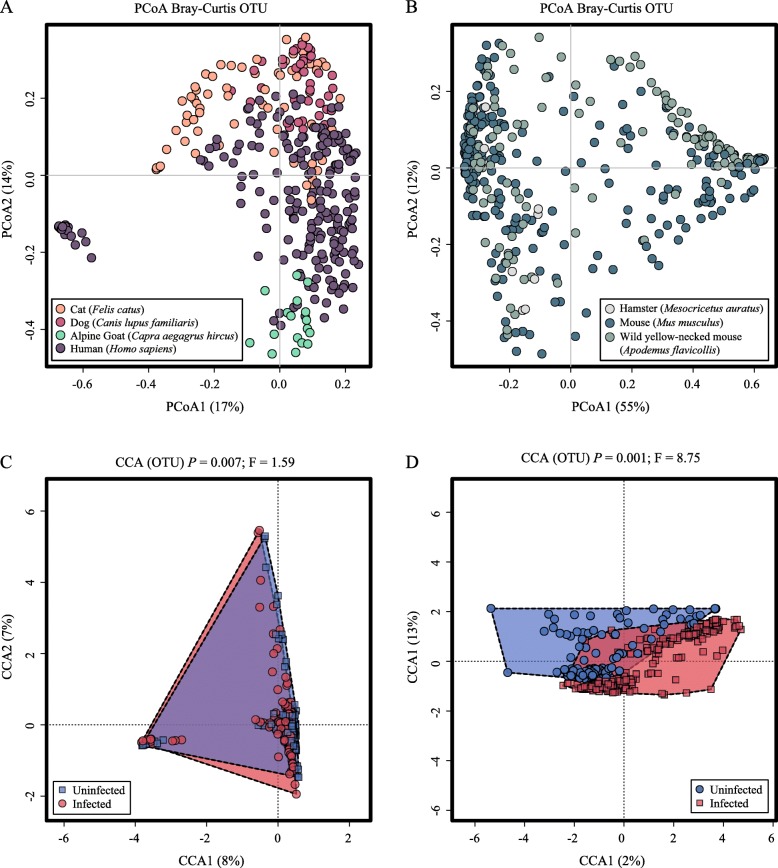
Gut microbial profiles of helminth-infected and -uninfected *Non*-*rodents* (i.e. human, cat, dog and goat) (**a**, **c**) and *Rodents* (i.e. mouse, hamster and wild yellow-necked mouse) (**b**, **d**), clustered at operational taxonomic unit (OTU) level by principal coordinates analysis (PCoA) (**a**, **b**), using ‘host’ as explanatory variable, and supervised canonical correspondence analysis (CCA) (**c**, **d**), using ‘infection status’ as explanatory variable

In order to assess the variability of the microbial community profiles included in our meta-analysis, individual sequence datasets generated from helminth-infected and -uninfected specimens were clustered at OTU level using PCoA, based on Bray-Curtis dissimilarity matrix and with ‘host’ as explanatory variable (Fig. 2). The two axes accounted for 44% of the total variance, i.e. 24% and 20% for PCoA1 and PCoA2, respectively (Fig. 2). In particular, the microbial profiles of specimens from humans, cats, dogs and goats clustered closely together, with minimal overlap with those from hamsters, mice and wild-mice (that were spread across the two variables) (Fig. 2). Therefore, given the largely distinct gut microbial profiles that characterised these two host groups, downstream analyses were conducted by categorising microbial profiles into ‘*Non*-*rodents*’ (i.e. human, cat, dog and goat, *n* = 339; helminth-uninfected *n* = 140 vs*.* -infected *n* = 199) and ‘*Rodents*’ (i.e. mouse, hamster and wild yellow-necked mouse, *n* = 393; helminth-uninfected *n* = 179 vs. infected *n* = 214) (cf. Fig. 3a, b). PCoA analyses conducted separately for each of the *Non*-*rodent* and *Rodent* microbial profiles revealed clustering according to specimen type (i.e. faeces or intestinal tissue) for the former, and according to collection site (i.e. stomach and small intestine, and large intestine and faeces) for the latter (cf. Additional file 1: Figure S1a and S1b). The microbial community profiles of both *Non*-*rodents* and *Rodents* were also segregated by hierarchical clustering and ordinated by supervised CCA, which revealed a significant association between microbiota composition and infection status (i.e. helminth-uninfected *vs*. -infected) for both *Non*-*rodents* (*p* = 0.007) and *Rodents* (*p* = 0.001) (Fig. 3c, d).

### Gut microbiota diversity indexes and differences in OTU abundance between helminth-uninfected and -infected hosts

**Fig. 4:**
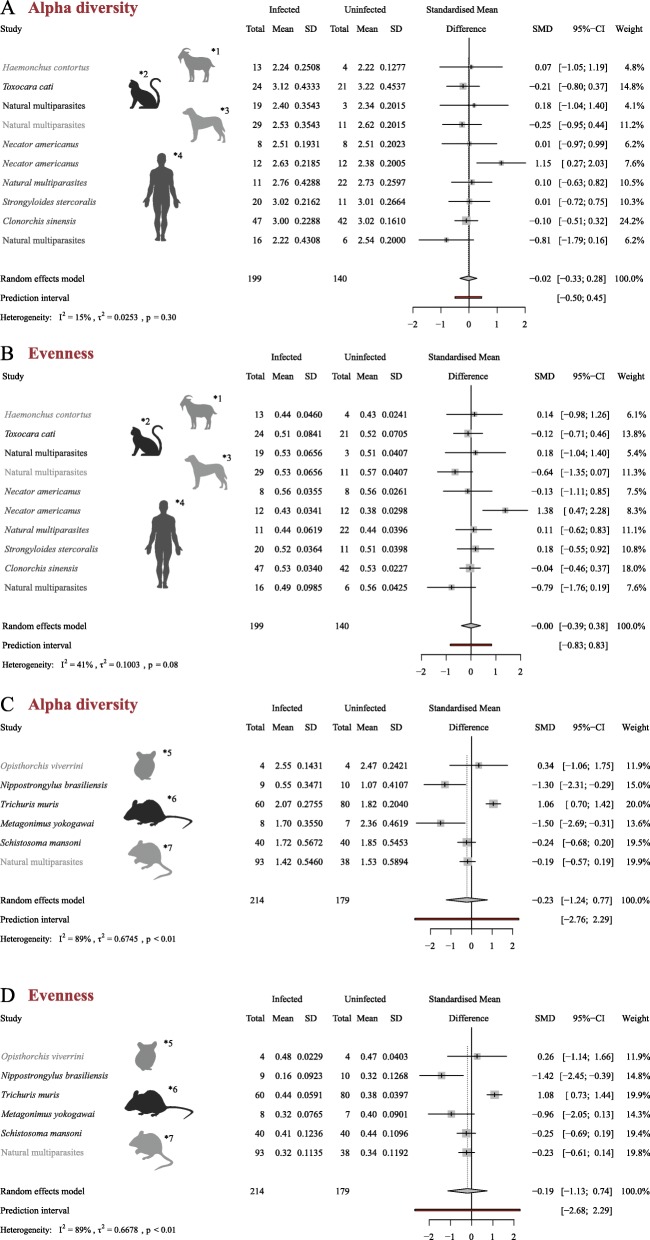
Gut microbial diversity of helminth-infected and -uninfected *Non*-*rodents* (i.e. human, cat, dog and goat) (**a**, **b**) and *Rodents* (i.e. mouse, hamster and wild yellow-necked mouse) (**c**, **d**). For each study included in the meta-analysis, the difference in gut microbial alpha diversity (Shannon index) (**a**, **c**) and evenness (**b**, **d**) between helminth-infected and –uninfected samples is shown, together with the overall estimate from the pooled random effect model (95% CI) of studies within each *Non*-*rodents* and *Rodents*. The random-effects meta-analysis model was performed using the DerSimonian–Laird estimator and adjusted using the Hartung-Knapp-Sidik-Jonkman method

For the *Non*-*rodents*, no significant differences in gut microbial alpha diversity were observed between helminth-infected and -uninfected hosts (standardized diversity difference [SMD] = − 0.02; standard deviation 95% confidence interval [95% CI] = [− 0.33; 0.28], random effects model pooled *p* = 0.30; Fig. 4a). For the *Rodents*, infection by parasitic helminths was associated with a decreased gut microbial alpha diversity (standardized Shannon index) when compared to the uninfected counterparts (standardized diversity difference [SMD] = − 0.23; standard deviation 95% confidence interval [95% CI] = [− 1.24; 0.77], random effects model pooled *p* < 0.01; Fig. 4c).

Similarly to alpha diversity, calculation of microbial species evenness (using Shannon Index) for the gut microbiota of helminth-infected and -uninfected *Non*-*rodents* yielded no significant differences between these two groups (standardized diversity difference [SMD] = 0.00; standard deviation 95% confidence interval [95% CI] = [− 0.39; 0.38], random effects model pooled *p* = 0.08; Fig. 4b); however, for *Rodents*, evenness was significantly lower in the gut microbiota of helminth-infected *vs*. -uninfected hosts (standardized diversity difference [SMD] = − 0.19; standard deviation 95% confidence interval [95% CI] = [− 1.13; 0.74], random effects model pooled *p* < 0.01; Fig. 4d).

**Fig. 5:**
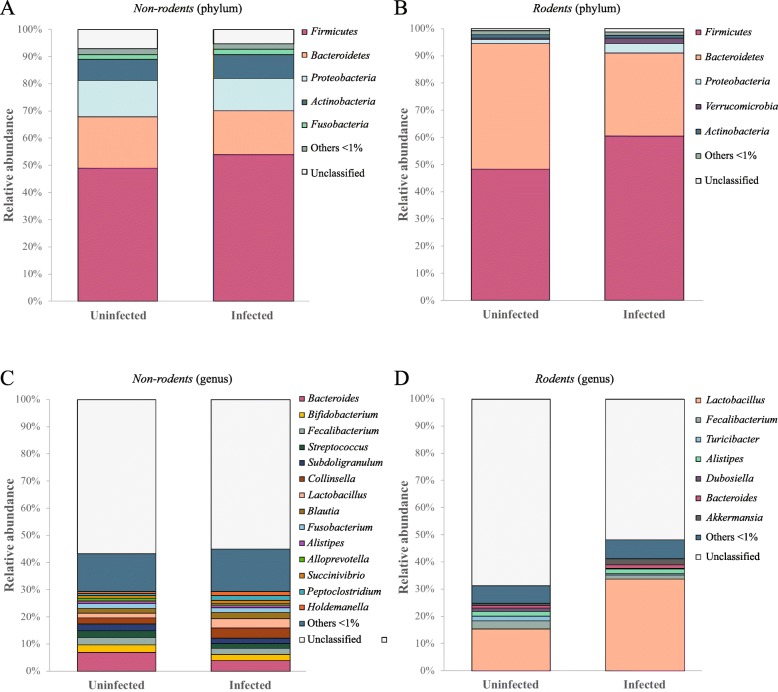
Microbial composition (at phylum and genus level) of helminth-infected and -uninfected samples from *Non*-*rodents* (**a**, **c**) and *Rodents* (**b**, **d**), ordinated according to infection status. Taxa making up < 1% of the overall microbiota are grouped under ‘unclassified’

Analysis of the relative abundances of gut microbial taxa of helminth-uninfected and -infected hosts in each *Non*-*rodents* and *Rodents* was performed at phylum and genus level using ‘infection status’ as explanatory variable (Fig. 5). For both groups, the phylum *Firmicutes* was predominant, and represented 48% of bacteria identified in helminth-uninfected samples, and 54% (*Non*-*rodents*) and 60% (*Rodents*) in helminth-infected samples, respectively (Fig. 5a, b). *Bacteroidetes* was the second most abundant group of bacteria in the gut microbiota of both groups, representing 18% and 16% of the microbial communities in helminth-uninfected and infected samples from the *Non*-*rodents*, and 46% and 30% of the remaining annotated OTUs in helminth-uninfected and -infected samples, respectively, in *Rodents* (Fig. 5a, b). RF classifier identified the phylum *Bacteroidetes* as the taxon discriminating the gut microbiota of helminth-uninfected and -infected hosts for both *Non*-*rodents* and *Rodents* (Table 2). In addition to *Bacteroidetes*, the phylum *Firmicutes* discriminated helminth-uninfected and -infected samples of *Rodents* with high MDA scores (i.e. 0.65) (Table 2). In the gut microbiota of *Non*-*rodents*, the remaining phyla identified included *Proteobacteria* (13% in helminth-uninfected *vs*. 12% in -infected samples) and *Actinobacteria* (7% in helminth-uninfected *vs*. 8% in -infected) (Fig. 5a). In the microbiota of *Rodents*, the remaining bacterial phyla identified were substantially less abundant (e.g. 3.5% *Proteobacteria* and 2.1% *Verrucomicrobia* in helminth-infected samples) (Fig. 5b). The latter phylum discriminated the gut microbial profiles of helminth-uninfected and -infected samples with high MDA scores (i.e. 0.58) (Table 2).

At genus level, 14 taxa (> 1% relative abundance) were identified in the gut microbiota of *Non*-*rodents*, with *Bacteroides* being predominant (i.e. 7% relative abundance in helminth-uninfected *vs*. 4% in -infected samples) (Fig. 5c). Seven bacterial genera were identified in the gut microbiota of *Rodents*, with the genus *Lactobacillus* making up 15% and 33% of the whole bacterial communities of helminth-uninfected and infected hosts, respectively (Fig. 5d). At genus level, *Bacteroides* discriminated between the gut microbiota of helminth-uninfected and -infected specimens in the *Non*-*rodents* (MDA = 0.60; Table 2), whereas the genera *Turicibacter* and *Lactobacillus* were significantly reduced and expanded in helminth-uninfected and -infected samples, respectively, from the *Rodents* (Table 2).

### Database properties and usage

A total of 54 published studies, spanning 33 parasites species and 14 vertebrate hosts, are currently stored in MICHELINdb (latest update October 2019). Each publication is assigned a unique ‘Mdb ID’ and an individual webpage including key study features (e.g. host and parasite species), sample metadata, microbiota profiling method, a summary of salient findings (as reported in the original publication) and details of microbial diversity indexes, as well as of specific taxonomic assignments of bacterial 16S rRNA sequences positively or negatively associated to helminth infection in the original publication (ranked according to phylum, order, class, family, genus and species). Links to the corresponding published article and to public sequence repositories hosting raw datafiles, as well as information on Open Access availability are also provided (see, for example, http://www.michelindb.com/a/d/c2f19866-eaf5-4b19-a7d5-95247f60f183).

MICHELINdb is searchable and browsable by several categories, including host and parasite species, microbiota profiling method and taxonomic classification of bacterial organisms with putative role(s) in helminth-microbiota interactions (according to available literature) by means of drop-down menus located at http://www.michelindb.com/a/as. In addition, metadata from individual studies can be exported by the user in .xml format. MICHELINdb will be subjected to regular updates (every 6 months) to ensure inclusion of new data and corresponding metadata from published investigations of helminth-microbiota relationships that were unavailable at the time of manuscript submission and peer-review, as well as post-website launch.

## Discussion

As a first step towards the identification of gut microbial taxa with potential roles in host-parasite crosstalk, we conducted a meta-analysis of 15 bacterial 16S rRNA sequence datasets obtained from seven host and ten parasite species, respectively, using a single bioinformatic pipeline. Such an approach allowed us to detect differences between the microbial profiles of rodent hosts (mainly laboratory models of helminth infections) and non-rodents (spanning experimentally and naturally helminth-infected humans and animals), which were therefore considered separately in downstream bioinformatic and biostatistical analyses of sequence data. The gut of rodent hosts has been reported to harbour a significantly different microbiome to that of humans and other mammalians [52, 53], which might explain the dissimilarities between the microbial profiles of these two groups observed in our meta-analysis. This observation calls for caution when translating findings obtained from mouse models of helminth infections to humans, and provide further support to the need of identifying common microbiota traits between laboratory rodents and ‘natural’ helminth hosts.

In addition, within each group of *Rodent* and *Non*-*rodent* hosts, the microbial profiles obtained from faecal samples were distinct from those obtained from individual GI compartments (cf. Additional file 3: Figure S1a and S1b). Faecal samples are often the sole accessible or available biological specimens for gut microbiota profiling for several hosts of helminth parasites [29] and therefore the use of the faecal microbiota as a proxy of GI microbial communities is necessary and often inevitable. However, inconsistencies have been detected between bacterial taxa whose relative abundances were correlated to helminth infection that were identified from faecal samples and gut luminal contents collected from the same vertebrate hosts [44, 54]. Indeed, subtle changes in the composition of the mucosally associated and/or luminal microbiota can be missed in stool samples [55]; similarly, variations in the abundances of bacterial taxa inhabiting regions of the GI tract downstream of the site of helminth localisation (mediated, for instance, by systemic immune responses mounted against the invading parasites) are unlikely to be detected in mucosal and/or luminal samples collected from the region of parasite establishment.

For each *Rodents* and *Non*-*rodents*, gut microbial communities formed two separate clusters according to helminth-colonisation status, thus providing support to a role of GI and EI parasite infections in shaping the gut bacterial make-up of infected hosts [20–22]. Of note, the pooling of 16S rRNA datasets generated from multiple studies allowed to detect statistically significant differences between the gut microbial profiles of helminth-infected and uninfected hosts that were previously unreported due to limitations in the numbers of specimens analysed in individual experiments [28, 56].

Comparative analyses of microbial alpha diversity between the gut microbiota of helminth-infected and -uninfected hosts yielded contrasting results between the *Non*-*rodents* and *Rodents*. In particular, for the *Non*-*rodents*, no significant changes in microbial alpha diversity were detected in gut bacterial communities according to infection status, with the exception of two studies conducted in human volunteers (cf. Fig. 4a, b) [44, 49]. The underlying characteristics of the individuals enrolled in these studies [52, 53], which differed substantially from those of other cohorts in the *Non*-*rodents*, may be responsible for the observed differences. Indeed, the study by Giacomin et al. [44] involved human subjects with pre-diagnosed Coeliac Disease experimentally infected with hookworm parasites (i.e. *N*. *americanus*) and subjected to increasing doses of gluten challenge, whilst Toro-Londono et al. [49] investigated the gut microbial profiles of children under 5 years of age infected by a GI protozoan (*Giardia*) and/or helminth parasites.

Analyses of differential abundance of bacterial taxa between the microbiota of helminth-infected and -uninfected hosts revealed the phylum *Bacteroidetes* as consistently reduced in the gut microbiota of helminth-colonised subjects (cf. Table 2, Fig. 5a, b). *Bacteroidetes* is one of the most abundant phyla of bacteria inhabiting the gut of vertebrates, with key roles in the metabolism of a wide range of carbohydrates [57]. In anaerobic environments, the products of fermentation of these substrates are short-chain fatty acids (SCFA) that are absorbed by the host gut and can act as source of ATP by the host cells [58]. In addition, SCFAs have been shown to interact with the host immune system, in particular by targeting G protein coupled receptors on intestinal epithelial cells and leukocytes and modulating their development, survival and function [59]. The reduction of populations of SCFA-producing bacteria in the gut microbiome of helminth-infected hosts included in our meta-analysis suggests that, whilst parasite-infections may be generally associated with decreased levels of microbial-derived SCFAs, the specific impact of parasite colonisation on the abundance of these compounds may depend on the helminth species under consideration (cf. [26]).

Conversely, bacteria belonging to the phylum *Firmicutes* were significantly more abundant in the gut microbiota of helminth-infected hosts when compared with that of the uninfected counterparts (cf. Table 2, Fig. 5a, b). In particular, in the *Rodents*, *Firmicutes* and *Verrucomicrobia* discriminated the microbial profiles of infected animals from those of uninfected controls. Within the *Firmicutes*, bacteria belonging to the genus *Lactobacillus* represented 15–33% of all genera of bacteria identified in the *Rodent* microbiota (Table 2 and Fig. 5d). Increased abundances in populations of lactobacilli have been repeatedly reported in the gut microbiota of rodent models of GI and EI helminth infections [23, 25, 28, 35, 60]; in addition, these bacteria have been suggested to represent key players in parasite-microbiota relationships by promoting the establishment of chronic infections in rodents colonised by the GI nematode *Heligmosomoides polygyrus* [23]. Nevertheless, thus far, similar lactobacilli abundances were observed in parasite-colonised and parasite-free human hosts [45, 46, 54, 61, 62]. Whether this discrepancy results from intrinsic differences between the host-parasite pairs under investigation, and/or from types of samples subjected to microbiota profiling (i.e. luminal content *vs*. stools) remains to be determined.

*Akkermansia* is a bacterial genus included within the phylum Verrucomicrobia. This genus (that includes the species *A*. *municiphila* and *A*. *glycaniphila*) was expanded in the gut microbiota of helminth-infected *Rodents* (Table 2 and Fig. 5d). These bacteria are known mucin-degrading anaerobes [63, 64] and their increased abundance may be directly linked to augmented mucin production by the host in response to parasite infection [65]. Indeed, mammalian mucins have been proposed to play a key role in the complex network of interactions occurring at the helminth-host interface [66], and increased mucin production has been linked to expanded populations of *Akkermansia* in the gut microbiota of macaques with chronic idiopathic diarrhoea infected with the whipworm *T*. *trichiura* [67]. Interestingly, the restoration of the gut barrier function mediated by the onset of Th2-type immunity stimulated by whipworm infection, and the consequent alterations in gut microbial composition, was hypothesised to represent one of the mechanisms by which these helminths were able to ameliorate the clinical signs of chronic inflammation in these animal models [67]. In contrast to *Akkermansia*, bacteria belonging to the genus *Turicibacter* (phylum Firmicutes) were consistently reduced in the gut microbiota of helminth-infected *Rodents*, although the role(s) that these microorganisms may play in the complex network of interactions between parasites and the resident gut microbiota, and/or in the pathophysiology of helminth infections remain(s) unclear [28].

Whilst this discussion focused on gut bacterial taxa whose relative abundances were repeatedly reported to be affected by helminth infections, several other bacterial groups that are seemingly unaffected by helminth colonisation may play yet undiscovered roles in host-parasite interactions. The application of shotgun metagenomic, metatranscriptomic and metaproteomic technologies to studies of the vertebrate gut microbiome in response to helminth infections may assist further investigations in this area; for instance, large-scale characterisations of gut bacterial gene expression prior to and following helminth colonisation could reveal key aspects of parasite-microbiota crosstalk that may not be reflected by significant changes in the relative abundances of individual microbial taxa post-helminth establishment.

## Conclusion

The (re)analysis of microbial 16S rRNA sequence data generated from a range of helminth-infected and -uninfected hosts and biological samples using a single bioinformatic pipeline allowed us to identify ‘common signatures’ characterising the microbial profiles of parasite-colonised hosts that may be targeted in future studies aimed at discovering and developing novel strategies of parasite treatment and control *via* the manipulation of resident populations of gut bacteria. Furthermore, we constructed MICHELINdb, a publicly available database including bacterial sequence data and sample metadata from published studies comparing the gut microbiota composition of several host species infected by one or multiple GI and/or EI helminth parasites with that of corresponding uninfected hosts. MICHELINdb allows easy data access, and enables time-efficient comparisons of quantitative and qualitative alterations in gut microbial composition upon GI and EI helminth infections detected in studies conducted in a range of host-parasite systems and using different experimental protocols and microbiota profiling techniques. Data and metadata linked to studies stored in MICHELINdb can be readily exported, to allow users to collect and store information, undertake further analyses and expand the meta-analysis dataset. Users are encouraged to submit newly generated data and metadata to MICHELINdb using the link provided, in order to enhance the exhaustiveness of the database and facilitate meaningful biological interpretations of study findings.

## Supplementary information

**Additional file 1: Table S1.** Studies investigating the relationships between helminth parasites and vertebrate gut microbiota included in the MICrobiome HELminth INteractions database (MICHELINdb). Studies from which datasets included in the meta-analysis were retrieved are indicated in green.

**Additional file 2: Table S2.** Metadata associated with individual helminth-infected and -uninfected samples (n = 732) included in the meta-analysis. Information provided was derived from the original publication or following personal communication with the corresponding author.

**Additional file 3: Figure S1.** Gut microbial profiles of helminth-infected and -uninfected samples. Samples from *Non-rodents* (i.e. human, cat, dog and goat) (**a**) and *Rodents* (i.e. mouse, hamster and wild yellow-necked mouse) (**b**), are clustered at operational taxonomic unit (OTU) level by supervised canonical correspondence analysis (CCA), using ‘type/site of sampling’ as explanatory variable.

## Data Availability

For each study included in the meta-analysis, raw sequence data and OTU assignments are available from the European Nucleotide Archive (ENA, https://www.ebi.ac.uk/ena) and from EBI MGnify (https://www.ebi.ac.uk/metagenomics/), respectively, under the corresponding ENA/MGnify accession reported in Table 1.
